# The Influence of Smoking on Pulmonary Tuberculosis in Diabetic and Non-Diabetic Patients

**DOI:** 10.1371/journal.pone.0156677

**Published:** 2016-06-07

**Authors:** Kuan-Jen Bai, Jen-Jyh Lee, Shun-Tien Chien, Chi-Won Suk, Chen-Yuan Chiang

**Affiliations:** 1 Division of Pulmonary Medicine, Department of Internal Medicine, Wan Fang Hospital, Taipei Medical University, Taipei, Taiwan; 2 School of Respiratory Therapy, College of Medicine, Taipei Medical University, Taipei, Taiwan; 3 Department of Internal Medicine, Buddhist Tzu Chi General Hospital and Tzu Chi University, Hualien, Taiwan; 4 Chest Hospital, Department of Health and Welfare, Tainan, Taiwan; 5 Department of Internal Medicine, School of Medicine, College of Medicine, Taipei Medical University, Taipei, Taiwan; 6 International Union Against Tuberculosis and Lung Disease, Paris, France; Barcelona University Hospital, SPAIN

## Abstract

**Background:**

Both smoking and diabetes can increase the risk and influence the manifestations and outcomes of tuberculosis (TB). It is not clear whether the influence of smoking on pulmonary TB differs between non-diabetic and diabetic patients. Herein, we assessed the manifestations and outcomes of TB in relation to smoking in both diabetic and non-diabetic TB patients.

**Methodology/Principal Findings:**

All diabetic culture-positive pulmonary TB patients notified from 2005–2010 at three teaching hospitals in Taiwan were enrolled. A culture-positive pulmonary TB patient without DM who was notified to the health authority immediately prior to each diabetic TB patient was selected for comparison. The 972 patients in this study cohort included 365 (37.6%) non-diabetic non-smokers, 149 (15.3%) non-diabetic smokers, 284 (29.2%) diabetic non-smokers, and 174 (17.9%) diabetic smokers. The adjusted relative risk of a pretreatment positive smear for a smoker compared with a non-smoker was 2.19 (95% CI 1.38–3.47) in non-diabetic patients and 2.23 (95% CI 1.29–3.87) in diabetic culture-positive pulmonary TB patients. The adjusted relative risk for a positive smear among diabetic smokers was 5.61 (95% CI 3.35–9.41) compared with non-diabetic non-smokers. Smoking was significantly associated with an increased frequency of bilateral lung parenchyma involvement (AdjOR 1.84, 95% CI 1.16–2.93), far-advanced pulmonary TB (AdjOR 1.91, 95% CI 1.04–3.50), cavitary lesions (AdjOR 2.03, 95% CI 1.29–3.20), and unfavorable outcomes of TB (AdjOR 2.35, 95% CI 1.02–5.41) in non-diabetic patients. However, smoking was not associated with cavitary lung parenchyma lesions regarding the location, number or size of the cavity in diabetic TB patients.

**Conclusions/Significance:**

Smoking and diabetes have joint effects on a pretreatment positive smear. Diabetic smokers had more than a 5-fold increased risk of a pretreatment positive smear than did non-diabetic non-smokers, indicating remarkable joint effects of diabetes and smoking on the risk of TB transmission.

## Introduction

Smoking is significantly associated with increased risks of tuberculous infection, tuberculosis (TB) disease, TB mortality and recurrent TB [1–5]. TB patients who have smoked are more likely to transmit TB to their child contacts [6]. A recent systematic review and meta-analysis reported that exposure to environmental tobacco smoke increases the risks of developing childhood TB disease and tuberculous infection [7]. Smoking influences the clinical manifestations and outcomes of TB. ‘Ever smokers’ were more likely to have experienced cough, dyspnea, cavity, miliary lung involvement, positive sputum culture and poor outcomes of TB [5, 8–11]. Diabetes mellitus (DM) is also associated with an increased risk of TB disease [12]. Diabetic TB patients have an increased risk of treatment failure, death and recurrent TB compared with non-diabetic TB patients [13, 14]. DM also influences the manifestations of TB. It has been reported that diabetic TB patients have more symptoms [15], are more likely to be smear-positive [16, 17], and have an increased frequency of cavitary lesions [18] compared with non-diabetic TB patients. Therefore, the World Health Organization (WHO) and the International Union Against Tuberculosis and Lung Disease (The Union) have encouraged National TB Programme to address the combined challenges of smoking, diabetes and TB [19, 20].

Although smoking and diabetes are both important risk factors for TB, it is unclear whether there is a differential influence of smoking on pulmonary TB between non-diabetic and diabetic patients. Previously, we have investigated the influence of diabetes on TB using representative samples of diabetic and non-diabetic culture positive pulmonary TB patients treated at three teaching hospitals in Taiwan [18, 21]. The smoking status of TB patients with or without diabetes was documented, which provided an opportunity to investigate joint effects and interactions between the influences of smoking and diabetes on TB. Herein, we report the manifestations and outcomes of pulmonary TB in association with smoking in both diabetic and non-diabetic culture positive pulmonary TB patients.

## Materials and Methods

### Study population

This present study was nested in a previous study of glycemic control and pulmonary TB, which was conducted at three teaching hospitals with respective locations in Northern, Eastern and Southern Taiwan [18, 21]. We obtained a list of all TB patients who were notified to health authorities from 2005–2010 and were managed by these three hospitals from the national TB registry at Taiwan CDC. Patients with DM were defined as those who 1) were treated with insulin or diabetes-specific hypoglycemic agents, 2) had been assigned an ICD-9 code related to DM during admission, 3) had been assigned an ICD-9 code related to DM two or more times on outpatient visits, or 4) had a history of DM. All diabetic culture-positive pulmonary TB patients who were notified from 2005–2010 were enrolled. For each diabetic culture-positive pulmonary TB patient, a culture-positive pulmonary TB patient without DM who was notified to the health authority immediately prior to the diabetic patient was selected for inclusion in a comparison group. As the non-diabetic culture-positive pulmonary TB patients were systematically selected, this was a representative sample of all non-diabetic culture-positive pulmonary TB patients who were treated at the three teaching hospitals from 2005–2010, which enabled us to assess the influence of smoking on pulmonary TB in relation to diabetes.

Among the 1209 culture-positive pulmonary TB patients (581 with DM and 628 without DM) who were included in the study of glycemic control and radiographic manifestations of pulmonary TB [18], 972 (80.4%; 649 non-smokers and 323 smokers) were included in this present study; 211 ex-smokers (a previous smoker who had stopped smoking prior to the diagnosis of TB), 21 patients whose smoking status was unknown, and 5 patients whose outcome was not available were excluded.

Clinical records were reviewed to determine patient age, sex, pretreatment smear (negative, positive, and positivity grade, scanty, 1+, 2+, 3+ 4+), type of TB case (new versus retreatment), smoking status, and treatment outcome. Patients were classified as a current smoker (those individuals who smoked at the time of TB diagnosis) or a non-smoker (those individuals who never smoked). Pretreatment drug susceptibility tests for isoniazid (H), rifampicin (R), ethambutol and streptomycin were collected and patients were classified as 1) susceptible, 2) showing resistance to H but not R (HrRs), 3) resistance to at least both H and R (HrRr), or 4) other resistance patterns. Data were collected from medical charts using a structured questionnaire.

The approach used to read chest x-rays has been reported previously [18]. Briefly, a pre-treatment postero-anterior chest radiograph was read by two qualified pulmonologists (readers) at each hospital. Readings were independent without discussion between the readers who were blinded to patients’ diabetic and smoking status. Films with any discordant reading were read by a third reader, who was a senior pulmonologist at each of the participating hospitals. Readings of the chest radiographs focused on lung parenchymal opacity and cavitation. Recordings of abnormal opacity of the lung parenchyma included location (right-upper, right-lower, left-upper, or left-lower) and the extent of disease (minimal, moderately-advanced, or far advanced). The extent of disease was estimated based on the sum of all areas of abnormality in which a boundary of abnormal opacity could be drawn. Minimal lesions were defined as an area less than that above a horizontal line across the 2^nd^ chondrosternal conjunction of one lung. Moderately advanced lesions were defined as an area greater in size than the minimal lesions but smaller than that of one entire lung. Far advanced lesions were defined as an area equivalent to or greater than one lung. The size of the largest cavity was dichotomized into small and large by the median diameter.

### Data entry and statistical analysis

To ensure the accuracy of data entry, the data set was “double entered” and validated using EpiData Entry 3.1 (EpiData Association, Odense, Denmark). Any discrepant records were checked and corrected against the original data on the questionnaires. STATA Version 12 (StataCorp LP, College Station, Texas, USA) was used for statistical analyses. A *P*-value <0.05 was considered to indicate a statistically significant difference.

We assessed the association between smoking and radiographic manifestations, symptoms, pre-treatment smears and smear positivity grades, as well as the outcome of TB for both diabetic and non-diabetic TB patients. Categorical data were analyzed using Pearson’s *χ*^2^ test. Logistic regression models were constructed for outcome variables with 2 categories, including the location of abnormal opacities and cavities, and the number and size of cavities; multinomial logistic regression models were constructed for outcome variables with 3 categories or more, including pretreatment smear findings (negative, positive, not done) and smear positivity grades (negative, low positivity grade [scanty, 1+, and 2+], high positivity grade [3+ and 4+]), and the extent of abnormal opacities (negative, minimal, moderately advanced, far advanced). In a treatment outcome analysis, outcomes were dichotomized into successful (treatment success) and unfavorable (died, failed, loss to follow-up). The association between smoking and patient outcomes was computed using multivariate logistic regression analysis, adjusted for age, sex, type of case, and anti-TB drug resistance. To assess whether diabetes is an effect modifier of smoking on TB, we performed analysis stratified by diabetes and regression models were constructed separately for diabetes and non-diabetic patients. Furthermore, regression models including a product term of smoking and diabetes were constructed for the total study population to assess interaction of diabetes and smoking.

### Ethics

This study was approved by the Joint Institute Review Board of Taipei Medical University. Written informed consent from each participant for his or her clinical records to be used in this study was waived. Patient information was anonymized and de-identified prior to analysis.

## Results

The cohort of 972 patients enrolled in this study included 365 (37.6%) non-diabetic non-smokers, 149 (15.3%) non-diabetic smokers, 284 (29.2%) diabetic non-smokers, and 174 (17.9%) diabetic smokers. Table 1 shows the characteristics of the 972 patients who were included in this study.

**Table 1:** Characteristics of the 972 culture-positive pulmonary tuberculosis patients included in this study, by diabetes and smoking.

	Non-Diabetes		Diabetes	
	Non smoker	Smoker	Non smoker	Smoker
Total	365	149	284	174
Sex				
Male	202 (55.3%)	135 (90.6%)	170 (59.9%)	166 (95.4%)
Female	163 (44.7%)	14 (9.4%)	114 (40.1%)	8 (4.6%)
Age (years)				
<45	124 (34.0%)	48 (32.2%)	22 (7.8%)	33 (19.0%)
45–64	91 (24.9%)	62 (41.6%)	127 (44.7%)	101 (58.1%)
≥65	150 (41.1%)	39 (26.2%)	135 (47.5%)	40 (23.0%)
Case type				
New	312 (85.5%)	128 (85.9%)	243 (85.6%)	157 (90.2%)
Retreatment	53 (14.5%)	21 (14.1%)	41 (14.4%)	17 (9.8%)
Smear				
Negative	171 (46.9%)	46 (30.9%)	93 (32.8%)	25 (14.4%)
Positive	157 (43.0%)	96 (64.4%)	174 (61.3%)	134 (77.0%)
Scanty	1 (0.3%)	1 (0.7%)	3 (1.1%)	4 (2.3%)
1+	47 (12.9%)	17 (11.4%)	38 (13.4%)	24 (13.8%)
2+	28 (7.7%)	24 (16.1%)	35 (12.3%)	22 (12.6%)
3+	26 (7.1%)	26 (17.5%)	47 (16.6%)	27 (15.5%)
4+	55 (15.1%)	28 (18.8%)	51 (18.0%)	57 (32.8%)
Not done	37 (10.1%)	7 (4.7%)	17 (6.0%)	15 (8.6%)
DST pattern*				
Susceptible	265 (72.6%)	110 (73.8%)	210 (73.9%)	133 (76.4%)
HrRs	23 (6.3%)	10 (6.7%)	19 (6.7%)	15 (8.2%)
HrRr	12 (3.3%)	6 (4.0%)	14 (4.9%)	5 (2.9%)
Other	7 (1.9%)	8 (5.4%)	8 (2.8%)	7 (4.0%)
Not done	58 (15.9%)	15 (10.1%)	33 (11.6%)	14 (8.1%)

* DST, drug susceptibility testing; Susceptible, susceptible to isoniazid, rifampicin, ethambutol, and streptomycin; HrRs, any resistance to isoniazid but not resistance to rifampicin; HrRr, resistance to at least both isoniazid and rifampicin; Other, other resistant patterns.

**Fig 1:**
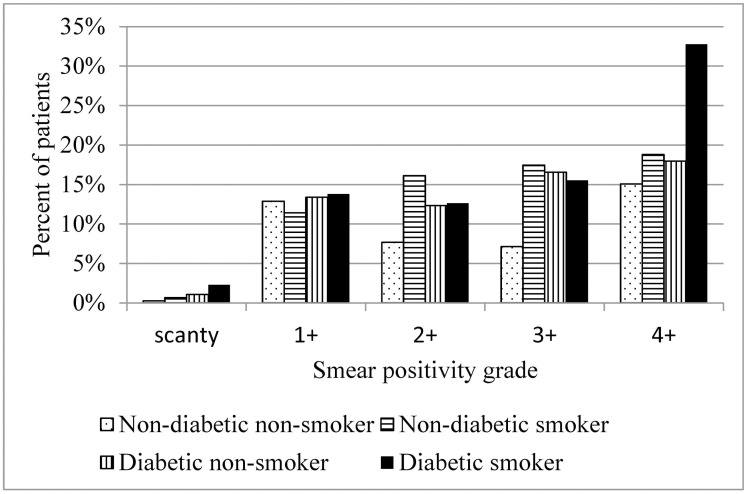
Pre-treatment smear positivity grades by smoking and diabetes.

The proportion of patients with a pretreatment positive smear was 43.0% in non-diabetic non-smokers, 64.4% in non-diabetic smokers, 61.3% in diabetic non-smokers, and 77.0% in diabetic smokers (*P*<0.001). The proportion of patients with high smear positivity grades (3+ or 4+) was 22.2% in non-diabetic non-smokers, 36.2% in non-diabetic smokers, 34.5% in diabetic non-smokers, and 48.3% in diabetic smokers (*P*<0.001; Fig 1). In a multinomial logistic regression analysis stratified by diabetes and adjusted for age and sex, the adjusted relative risk of pretreatment positive smears for smokers compared with non-smokers was 2.19 (95% CI 1.38–3.47) in non-diabetic and 2.23 (95% CI 1.29–3.87) in diabetic culture-positive pulmonary TB patients. In a multinomial logistic regression analysis of the whole study population without stratification and including a product term of diabetes and smoking, there was no significant interaction of smoking and diabetes for pretreatment positive smear (p = 0.517). The relative risk for a pretreatment positive smear among diabetic smokers was more than 5-fold higher than for non-diabetic non-smokers (AdjRRR 5.61, 95% CI 3.35–9.41). Smoking was also significantly associated with a high (3+ or 4+) smear positivity grade in both non-diabetic (AdjRRR 2.32, 95% CI 1.36–3.98) and diabetic (AdjRRR 2.29, 95% CI 1.26–4.15) culture-positive pulmonary TB patients in a multinomial logistic regression analysis stratified by diabetes and adjusted for age and sex (Table 2). In a multinomial logistic regression analysis of the whole study population without stratification and including a product term of diabetes and smoking, there was no significant interaction of smoking and diabetes for a pretreatment high smear positivity grade (p = 0.546). The relative risk for a pretreatment high smear positivity grade among diabetic smokers was more than 6-fold higher than for non-diabetic non-smokers (AdjRRR 6.60, 95% CI 3.74–11.66). (Table 2)

**Table 2:** Pretreatment-positive sputum smears and high smear positivity grades (3+ or 4+) in relation to smoking in culture-positive pulmonary tuberculosis patients at three tertiary referral hospitals in Taiwan, 2005–2010, stratified by diabetes.

			Multivariate analysis*			
			Overall		Stratified by diabetes	
	N	%	AdjRRR	95% CI	AdjRRR	95% CI
**Smear positive**						
Non-diabetes						
Non-smoker	157	43.0	1		1	
Smoker	96	64.4	2.08	1.33–3.25	2.19	1.38–3.47
Diabetes						
Non-smoker	174	61.3	2.19	1.53–3.13	1	
Smoker	134	77.0	5.61	3.35–9.41	2.23	1.29–3.87
**High positivity grade**						
Non-diabetes						
Non-smoker	81	22.2	1		1	
Smoker	54	36.2	2.17	1.29–3.64	2.32	1.36–3.98
Diabetes						
Non-smoker	98	34.5	2.43	1.60–3.70	1	
Smoker	84	48.3	6.60	3.74–11.66	2.29	1.26–4.15

*Adjusted relative risk ratio, adjusted for age group and sex; 95% CI, 95% confidence interval.

Table 3 shows the location and extent of abnormal opacities in the lung parenchyma on chest radiographs in relation to smoking in culture-positive non-diabetic and diabetic pulmonary TB patients. Smoking was associated with an increased frequency of bilateral lung parenchyma involvement (AdjOR 1.84, 95% CI 1.16–2.93) and far-advanced pulmonary TB (AdjOR 1.91, 95% CI 1.04–3.50) in non-diabetic TB patients. However, smoking was not found to be associated with lung parenchyma lesions in terms of the location or extent of disease in diabetic TB patients.

**Table 3:** Location and extent of abnormal opacities of the lung parenchyma on chest radiographs in relation to smoking in culture-positive pulmonary tuberculosis patients at three tertiary-referral hospitals in Taiwan, 2005–2010, stratified by diabetes.

	Non-smoker		Smoker		Multivariate		
	No.	%	No.	%	AdjOR	95% CI	
**Non-diabetic**	365	100	149	100			
Any opacity	357	97.8	146	98.0	1.09	0.29	4.17
**Location**							
Upper field	321	88.0	143	96.0	2.01	0.77	5.30
Lower field	228	62.5	106	71.1	1.57	0.98	2.50
Right field	299	81.9	133	89.3	1.76	0.93	3.36
Left field	262	71.8	121	81.2	1.51	0.89	2.54
Bilateral field	204	55.9	108	72.5	1.84	1.16	2.93
**Extent**							
Minimal	157	43.0	42	28.2	Base		
Moderately-advanced	149	40.8	69	46.3	1.48	0.90	2.42
Far-advanced	51	14.0	35	23.5	1.91	1.04	3.50
**Diabetic**	284	100	174	100			
Any opacity	282	99.3	171	98.3	0.40	0.07	2.44
**Location**							
Upper field	267	94.0	164	94.3	0.87	0.33	2.30
Lower field	209	73.6	133	76.4	0.98	0.59	1.61
Right field	235	82.8	142	81.6	0.88	0.50	1.56
Left field	222	78.2	137	78.7	0.77	0.45	1.32
Bilateral field	175	61.6	108	62.1	0.86	0.55	1.35
**Extent**							
Minimal	84	29.6	38	21.8	Base		
Moderately-advanced	149	52.5	90	51.7	1.02	0.60	1.73
Far-advanced	49	17.3	43	24.7	1.64	0.86	3.13

Note: No, number; CI, confidence interval; AdjOR, adjusted odds ratio, adjusted for sex and age group.

Table 4 shows the location and size of cavitary lesions in the lung parenchyma on a chest radiograph in relation to smoking in culture-positive non-diabetic and diabetic pulmonary TB patients. Smoking was found to be significantly associated with an increased frequency of cavitary lesions (AdjOR 2.03, 95% CI 1.29–3.20) in non-diabetic pulmonary TB patients; it mainly affected the upper-lung field (AdjOR 2.25, 95% CI 1.42–3.57), but not the lower lung field (AdjOR 0.67, 95% CI 0.23–1.93). Smoking was also significantly associated with an increased frequency of multiple cavities (AdjOR 1.88, 95% CI 1.13–3.14) and large (≥3 cm) cavities (AdjOR 2.03, 95% CI 1.29–3.20) in non-diabetic TB patients. However, smoking was not associated with cavitary lung parenchyma lesions in terms of the location, number, or size of cavitary lesions in diabetic TB patients.

**Table 4:** Location and size of cavitary lesions in the lung parenchyma on chest radiographs in relation to smoking in culture-positive pulmonary tuberculosis patients at three tertiary-referral hospitals in Taiwan, 2005–2010, stratified by diabetes.

	Non-smoker		Smoker		Multivariate		
	No.	%	No.	%	AdjOR	95% CI	
**Non-diabetes**	365	100	149	100			
Any cavity	86	23.6	68	45.6	2.03	1.29	3.20
**Location**							
Upper field	79	21.6	67	45.0	2.25	1.42	3.57
Lower field	15	4.1	6	4.0	0.67	0.23	1.93
Right field	48	13.2	48	32.2	2.35	1.40	3.93
Left field	58	15.9	45	30.2	1.69	1.02	2.81
Bilateral field	20	5.5	25	16.8	2.43	1.21	4.89
**Number and size**							
Multiple	51	14.0	45	30.2	1.88	1.13	3.14
Large (≥3 cm)	35	9.6	35	23.5	2.03	1.29	3.20
**Diabetes**	284	100	174	100			
Any cavity	126	44.4	105	60.3	1.20	0.76	1.88
**Location**							
Upper field	113	39.8	98	56.3	1.28	0.82	2.00
Lower field	36	12.7	29	16.7	0.91	0.49	1.67
Right field	74	26.1	61	35.1	0.91	0.57	1.46
Left field	74	26.1	64	36.8	1.23	0.76	1.98
Bilateral field	22	7.8	20	11.5	0.82	0.40	1.68
**Number and size**							
Multiple	70	24.7	69	39.7	1.21	0.75	1.95
Large (≥3 cm)	55	19.4	57	32.8	1.20	0.76	1.88

Note: No, number; CI, confidence interval; AdjOR, adjusted odds ratio, adjusted for sex and age group.

Among the 972 TB patients, 885 (91.1%) were successfully treated, 63 (6.5%) died, 9 (0.9%) were lost to follow-up, and 15 (1.5%) failed. Table 5 shows that smoking was significantly associated with an unfavorable treatment outcome in non-diabetic pulmonary TB patients (*P* = 0.032), and was associated with a higher frequency of death (6.7% vs. 3.8%) and failure (2.7% vs. 0.6%) among smokers compared with non-smokers. However, smoking was not significantly associated with an unfavorable outcome for TB among diabetic pulmonary TB patients (*P* = 0.407). In a multivariate logistic regression analysis of the whole study population without stratification that adjusted for age, sex, type of case, anti-TB drug resistance, there was significant negative interaction of smoking and diabetes(p = 0.013) and the effect of smoking on outcome of TB was attenuated by diabetes. However, we found that non-diabetic smokers (AdjOR 3.12, 95% CI 1.40–6.98), diabetic non-smokers (AdjOR 3.32, 95% CI 1.75–6.31), and diabetic smokers (AdjOR 2.95, 95% CI 1.31–6.66) were significantly associated with increased risks of unfavorable outcomes for TB treatment compared with non-diabetic non-smokers (Table 6). When stratified by diabetes, smoking was found to be significantly associated with an unfavorable outcome for TB treatment in non-diabetic TB patients (AdjOR 2.35, 95% CI 1.02–5.41), but did not increase the risk of an unfavorable outcome among diabetic TB patients (AdjOR 1.14, 95% CI 0.55–2.37).

**Table 5:** Outcomes of treatment in relation to smoking in culture-positive pulmonary tuberculosis patients at three tertiary-referral hospitals in Taiwan, 2005–2010, stratified by diabetes.

	Success		Died		Lost		Failed		*P*-value
	N	%	N	%	N	%	N	%	
Non-diabetes									0.032
Non-smoker	349	95.6	14	3.8	0	0	2	0.6	
Smoker	134	89.9	10	6.7	1	0.7	4	2.7	
Diabetes									0.407
Non-smoker	244	85.9	29	10.2	5	1.76	6	2.1	
Smoker	158	90.8	10	5.8	3	1.7	3	1.7	

Note: treatment success (documented sputum culture conversion and remained culture negative till completion of a treatment course); failed (sputum culture positive at 5 months of treatment or later); lost-to-follow-up (interruption of treatment for 2 consecutive months or lack of outcome assessment); died (died of any cause during TB treatment)

**Table 6:** Smoking and unfavorable tuberculosis outcomes (died, loss-to-follow-up, or failed) in the study population, stratified by diabetes.

	Total	Unfavorable		Multivariate analysis			
				Overall		Stratified by diabetes	
		N	%	AdjOR*	95% CI	AdjOR*	95% CI
Non-diabetes							
Non-smoker	365	16	4.4	1		1	
Smoker	149	15	10.1	3.12	1.40–6.98	2.35	1.02–5.41
Diabetes							
Non-smoker	284	40	14.1	3.32	1.75–6.31	1	
Smoker	174	16	9.2	2.95	1.31–6.66	1.14	0.55–2.37

*Adjusted for age group, sex, type of case (new vs. previously treated) and drug resistance.

## Discussion

Our present study shows that the influence of smoking on pulmonary TB is different between non-diabetic and diabetic patients. Smoking was found to be significantly associated with an increased frequency of bilateral lung parenchyma involvement, far-advanced pulmonary TB, cavitary lesions, multiple cavities, large cavities, and an unfavorable outcome of TB treatment in non-diabetic, but not in diabetic culture-positive pulmonary TB patients because the effect of smoking was attenuated by diabetes. However, smoking was found to be associated with an increased frequency of pretreatment-positive smears in both non-diabetic and diabetic culture-positive pulmonary TB patients and the effect was not attenuated by diabetes.

An association between smoking and the manifestations of TB has been previously reported in several publications, although these findings have not been consistent. Leung et al assessed 851 TB patients and reported that ‘ever smokers’ were more likely to have upper zone involvement (OR 1.67), cavities (OR 1.76), miliary lung involvement (OR 2.77), and positive sputum cultures (OR 1.43) [8]. Altet-Gómez et al assessed 8903 pulmonary TB patients in whom 35.8% had cavitary lesions on CXR and 66.2% were smear positive; smokers were more likely to have cavitary lesions (OR 2.2, 95% CI 2.0–2.4%) and to be smear positive (OR 2.0, 95% CI 1.8–2.3%) than non-smokers [9]. Wang investigated 523 patients and reported that ‘ever smokers’ were more likely to have upper-lung predominance and cavitation compared with ‘never smokers’, but there was no significant difference in the pretreatment positive smears between ‘ever smokers’ and ‘never smokers’ [10]. Leung et al investigated 16,345 TB patients in Hong Kong and reported that smoking was associated with more extensive lung disease, lung cavitation, and positive sputum smears (29.2% among non-smokers, 39.6% among ex-smokers, and 36.7% among smokers) and culture results at the baseline [5].

In our present study, the proportion of patients with upper-lung field involvement was higher among smokers than non-smokers in non-diabetic patients, although the difference did not meet our threshold for statistical significance. Our findings confirm the finding that smoking is associated with extensive lung disease and cavitary lesions, but these were only observed in non-diabetic and not in diabetic TB patients. Several studies have reported that diabetes is associated with cavitary lesions on CXR [14, 16–18, 22, 23]. Our study reveals that the effect of smoking on cavitary lesions or far advanced lesions on CXR was attenuated by diabetes.

The most striking finding of our study was that smoking and diabetes showed joint effects on pretreatment positive smears: smokers had more than a 2-fold increased risk of pretreatment positive smears in both non-diabetic and diabetic TB patients and diabetic smokers had more than a 5-fold increased risk of pretreatment positive smears than did non-diabetic non-smokers. Furthermore, the increased frequency in positive smears was mainly of a high positivity grade, indicating remarkable joint effects of diabetes and smoking that increased the risk of transmission of TB. This finding implies that radiographic manifestations may not be sufficiently sensitive to capture the joint effects of diabetes and smoking on the bacillary load of TB.

Regarding outcomes of TB, Wang et al reported that smoking was not associated with a poor prognosis for TB in a multi-variate survival analysis [10]. Leung et al reported that the proportion of patients with treatment success was 76.7% among ex-smokers and 81.5% among smokers, which were both lower than the 84.7% among non-smokers as a consequence of the higher proportion of death among ex-smokers and current smokers [5]. Reed et al reported that the risk of death in the first 12 months of enrollment was 2.2-fold higher than in non-diabetic patients and that the risk of death among diabetic smokers who smoked one or more pack of cigarettes daily prior to enrollment was 4.3-fold higher than for non-diabetic non-smokers [24]. Our study confirmed that smoking was significantly associated with an unfavorable outcome of TB, but the increased risk of unfavorable outcome occurred only in non-diabetic patients. In our present study, diabetes was associated with an increased risk of an unfavorable outcome of TB. The effect of smoking was attenuated by diabetes and smoking was not associated with an unfavorable outcome of TB in diabetic patients.

Our study has several limitations. First, we found that smoking was significantly associated with extensive disease, cavitary lesions, and unfavorable outcomes of TB in non-diabetic, but not in diabetic culture-positive pulmonary TB patients. The sample size of our study may not have been sufficient to detect the influence of smoking on the radiographic manifestations and outcomes of pulmonary TB in diabetic patients. Second, we did not have data regarding the daily use of cigarettes, so we could not assess the dose response relationship between smoking and TB. Third, several studies have reported the underlying mechanisms of the impact of smoking on TB [25, 26]. Our present study had an observational design, which by nature did not allow us to investigate how smoking and diabetes jointly modify the innate and adaptive immune responses to TB. Fourth, the study is a retrospective study based on charts review. There might be mis-classification of smoking status of TB patients. However, mis-classification of exposure tends to bias results toward the null. Thus, our analysis may under-estimate the influence of smoking on TB.

Nevertheless, our study clearly established that smoking is significantly associated with an increased frequency of bilateral lung parenchyma involvement, far-advanced pulmonary TB, cavitary lesions, multiple cavities, large cavities, and unfavorable outcomes of TB treatment in non-diabetic TB patients. Furthermore, smoking and diabetes have joint effects on pretreatment-positive smears and a high proportion of smokers and diabetic patients have high smear positivity grades. Therefore, the benefit of tobacco control on TB responses should be noted [27]. The ‘MPOWER’ package developed by the WHO to address the global tobacco epidemic includes the following suggestions: **M**onitor tobacco use and prevention policies, **P**rotect people from tobacco smoke, **O**ffer help to quit tobacco use, **W**arn about the dangers of tobacco, **E**nforce bans on tobacco advertising, promotion and sponsorship, and **R**aise taxes on tobacco. Levy et al reported that in 41 countries that adopted at least one of the highest-level MPOWER policies between 2007 and 2010, the number of smokers was estimated to have dropped by 14.8 million [28]. Moreover, several studies have reported that even briefly providing advice regarding smoking cessation among TB patients could result in a high quit rate among TB patients who smoked [29–32]. The findings of our present study provide additional evidence to highlight the potential contributions of tobacco control on TB control.

## Supporting Information

S1 Data Dataset underlying the findings in the manuscript. (DTA)
